# Mapping Pathological Phenotypes in Reelin Mutant Mice

**DOI:** 10.3389/fped.2014.00095

**Published:** 2014-09-04

**Authors:** Caterina Michetti, Emilia Romano, Luisa Altabella, Angela Caruso, Paolo Castelluccio, Gaurav Bedse, Silvana Gaetani, Rossella Canese, Giovanni Laviola, Maria Luisa Scattoni

**Affiliations:** ^1^Neurotoxicology and Neuroendocrinology Section, Department of Cell Biology and Neuroscience, Istituto Superiore di Sanità, Rome, Italy; ^2^Department of Physiology and Pharmacology “V. Erspamer”, Sapienza University of Rome, Rome, Italy; ^3^Behavioural Neuroscience Section, Department of Cell Biology and Neuroscience, Istituto Superiore di Sanità, Rome, Italy; ^4^Bambino Gesù Children’s Hospital, Istituto Di Ricovero e Cura a Carattere Scientifico, Rome, Italy; ^5^Molecular and Cellular Imaging Section, Department of Cell Biology and Neuroscience, Istituto Superiore di Sanità, Rome, Italy; ^6^Department of Psychology, School of Behavioural Neuroscience, Sapienza University of Rome, Rome, Italy

**Keywords:** autism spectrum disorders, reeler mice, ultrasonic vocalizations, social interaction, stress response, dopamine, glutamate, circadian cycle

## Abstract

Autism Spectrum Disorders (ASD) are neurodevelopmental disorders with multifactorial origin characterized by social communication deficits and the presence of repetitive behaviors/interests. Several studies showed an association between the *reelin* gene mutation and increased risk of ASD and a reduced reelin expression in some brain regions of ASD subjects, suggesting a role for *reelin* deficiency in ASD etiology. Reelin is a large extracellular matrix glycoprotein playing important roles during development of the central nervous system. To deeply investigate the role of *reelin* dysfunction as vulnerability factor in ASD, we assessed the behavioral, neurochemical, and brain morphological features of reeler male mice. We recently reported a genotype-dependent deviation in the ultrasonic vocal repertoire and a general delay in motor development of reeler pups. We now report that adult male heterozygous (Het) reeler mice did not show social behavior and communication deficits during male–female social interactions. Wildtype and Het mice showed a typical light/dark locomotor activity profile, with a peak during the central interval of the dark phase. However, when faced with a mild stressful stimulus (a saline injection) only Het mice showed an over response to stress. In addition to the behavioral studies, we conducted high performance liquid chromatography and magnetic resonance imaging and spectroscopy to investigate whether *reelin* mutation influences brain monoamine and metabolites levels in regions involved in ASD. Low levels of dopamine in cortex and high levels of glutamate and taurine in hippocampus were detected in Het mice, in line with clinical data collected on ASD children. Altogether, our data detected subtle but relevant neurochemical abnormalities in reeler mice supporting this mutant line, particularly male subjects, as a valid experimental model to estimate the contribution played by *reelin* deficiency in the global ASD neurobehavioral phenotype.

## Introduction

Autism Spectrum Disorders (ASD) are neurodevelopmental disorders with multifactorial origin characterized by persistent deficits in social communication and interaction and restricted and repetitive patterns of behavior, interests, or activities (1). Several studies showed that abnormal reelin expression in the brain is involved in a number of neuropsychiatric disorders including lissencephaly, schizophrenia, and autism (2–8).

Clinical studies have shown reduced levels of reelin protein in blood serum and in post-mortem brain of ASD patients (9–12). Genetic variants in RELN have been investigated as risk factors of ASD in numerous epidemiologic studies but with inconclusive results (13–19). However, recent data collected on much larger samples and with more advanced genetic approaches indicated a relationship between *reelin* gene mutation and increase risk of autism, suggesting that *reelin* deficiency may be a vulnerability factor in the etiology of this neurodevelopmental disorder (20–27).

Animal models in which reelin expression is reduced or absent, provide important information about the role of *reelin* deficiency in the onset of neurodevelopmental disorders such as ASD. Homozygous reeler mice show decreased brain volume, increased ventricles volume, (28–30), a non-foliated cerebellum (30), reduced number of Purkinje cells (31), deficits in lamination of the hippocampus (Hip), and disorganization of the amygdala (30). Some of these abnormalities are comparable with the ones found in post-mortem studies on autistic brain such as: increased ventricle volume, altered cortical lamination, heterotopias, dysplastic changes, and reduced number of Purkinje cells (32–39). These morphological changes in homozygous reeler mice are also associated with serious physical impairments and for this reason these mice are not considered as a reliable animal model for basic behavioral research but their use has been so far limited to the study of neuronal migration and of etiology of human lissencephaly (4, 5).

Heterozygous reeler mice, which exhibit the 50% reduction in reelin expression, do not display a reeler phenotype but express a number of abnormal traits including loss of Purkinje cells of the cerebellum (40, 41) and decrease in the number of dendritic spines in cortical and hippocampal neurons (42). Reduced levels of reelin are also associated with an increased anxiety profile (43, 44), cognitive deficits in the operant conditioning (44, 45), executive functions (46), fear conditioning learning (47, 48), olfactory conditioning learning (49), latent inhibition (50), and attentional set-shifting (51).

Surprisingly, only limited studies have investigated the contribution of *reelin* deficiency to the establishment of the social/communicative deficits, first ASD core symptom as indicated in the DSM 5 (1). Adult social responses in heterozygous (Het) reeler mice have been tested so far in two studies assessing either direct male–male and female–female social interactions (52) or performance in a modified version of the three-chamber sociability test (51). In both studies, only social behavioral performances have been assessed but a detailed evaluation of the ultrasonic vocalizations (USVs) emitted during the interaction was missing. To this aim, we deeply investigated the social and vocal repertoire of wildtype (Wt) and Het reeler mice during courtship (53), to evaluate the presence of qualitative alterations in social interaction and communication in this mutant line. In addition, we evaluated the baseline circadian locomotor activity in the home-cage as well as the response to a mild stressful stimulus represented by a saline injection (54–57) to check for abnormalities in the spontaneous locomotor activity that could affect the behavioral performances. To investigate whether *reelin* mutation influences brain metabolism, brain morphology, and levels of monoamines and their metabolites into selected brain regions involved in ASD and social behavior, we performed *in vivo* quantitative magnetic resonance imaging (MRI), spectroscopy, and high performance liquid chromatography (HPLC) analyses.

## Materials and Methods

### Animals and housing

Breeding pairs were originally purchased from The Jackson Laboratory (Bar Harbor, ME, USA) and bred in our laboratory at ISS. About 2 weeks after pairing for breeding (15 Het × Het crosses), the females were individually housed and subsequently inspected daily for pregnancy and delivery. After weaning on postnatal day (pnd) 25, mice were housed by sex in mixed genotype groups (B6C3Fe Wt and Het) of two to three per cage. All mice were housed in a colony room with temperature maintained at 21 ± 1°C and humidity at 60 ± 10% with food (Enriched standard diet purchased from Mucedola, Settimo Milanese, Italy) and water available *ad libitum*. The colony room was maintained on a 12:12 light/dark cycle with lights on at 18.30 h. Mice genotype was determined at pnd 21 by polymerase chain reaction (PCR) analysis on tale samples and the animals were marked by an ear punching for identification. Consistent with the higher prevalence of autism in human males, only male mice were tested. Homozygous reeler mice were not tested due to their serious physical impairments after weaning. The same cohort of adult male mice was tested for male–female reciprocal social interaction with concomitant USVs (3 months of age), locomotor activity in the home-cage (6 months), and HPLC (7 months). A separate cohort of mice was subjected to *in vivo* quantitative MRI and spectroscopy at 4 months of age. All procedures were conducted in strict compliance with the European Communities guidelines (EC Council Directive 86/609), Italian legislation on animal experimentation (Decreto L.Vo 116/92).

### Adult male–female social interactions

Three-month-old male mice (*N* = 9 Wt, *N* = 21 Het) were evaluated in the male–female social interaction test as in Ref. (53). Each male subject was isolated 1 h before testing and the vaginal estrous condition of each stimulus female was assessed as in Ref. (58). Only females in estrous were selected for the test. The unfamiliar stimulus C57BL/6J female mouse was placed into the home-cage of the isolated male mouse and behaviors and USVs were recorded for a 3-min test session. Stimulus mice (C57BL/6J females) were purchased from Jackson Laboratories (Bar Harbor, ME, USA) and maintained in our colony room in social groups of three per home-cage. Each female was used only twice and were matched to the subject mice by age and body weight.

Social interaction test was conducted between 09.00 and 13.00 h, during the dark phase, under red light. In addition to the isolated mouse, the cage contained litter (1.5-cm deep) and the lid was removed during the test. For video recordings, the videocamera (Panasonic monochrome charge-coupled device camera) was mounted facing the side of the cage and the subsequent scoring of social investigation parameters was conducted with Noldus Observer 10XT software (Noldus Information Technology, Leesburg, VA, USA).

Social interactions were scored from the videotapes for the frequencies and durations of the following behavioral responses performed by the subject mouse: *anogenital sniffing* (direct contact with the anogenital area), *body sniffing* (sniffing or snout contact with the flank area), *head sniffing* (sniffing or snout contact with the head/neck/mouth area), *locomotor activity*, *rearing* up against the wall of the home-cage, *digging* in the bedding, and *grooming* (self-cleaning, licking any part of its own body). No observations of *mounting*, *fighting*, *tail rattling*, and *wrestling* behaviors were observed. Scoring was conducted by two investigators uninformed of the genotype. Inter-rater reliability was 98%.

For audio recordings, the ultrasonic microphone (Avisoft UltraSoundGate condenser microphone capsule CM16, Avisoft Bioacoustics, Berlin, Germany) was mounted 20 cm above the cage and the USVs recorded using Avisoft RECORDER software version 3.2. Settings included sampling rate at 250 kHz; format 16 bit. The ultrasonic microphone was sensitive to frequencies between 10 and 180 kHz. For acoustical analysis, recordings were transferred to Avisoft SASLabPro (version 4.40) and a fast Fourier transformation (FFT) was conducted as previously described (59). Start times for the video and audio files were synchronized. Parameters analyzed included number and mean duration of calls, qualitative and quantitative analyses of sound frequencies measured in terms of frequency, and amplitude at the maximum of the spectrum. Waveform patterns of calls [a total of 17195 (Wt) and 8454 (Het) calls] were examined in depth in the sonograms collected from every mouse tested. Each call was identified as one of eight distinct categories, based on internal pitch changes, lengths, and shapes, as in our previously published studies (53, 59, 60).

Inter-rater reliability in scoring the call categories was 98%. Scoring was conducted by two investigators blind to the mouse genotype. Call category data were subjected to two different analyses: (1) Genotype-dependent effects on the probability of producing calls (proportion of calls in each category for each subject) from each of the eight categories of USV, as described below under statistical analysis; (2) a descriptive analysis that included genotype-dependent effects on the percentage of calls emitted by each subject in each of the eight categories of USV.

### Locomotor activity in the home-cage

At 6 months of age, male mice (*N* = 9 Wt, *N* = 10 Het) were individually housed in standard cages (33 cm × 13 cm × 14 cm) and assigned to a continuous monitoring of spontaneous locomotor activity. The assessment of daily spontaneous activity in the home-cage was carried out by means of an automatic device using small passive infrared sensors positioned on the top of each cage (Activiscope system, see the website: www.newbehavior.com) (61–63). The system operated continuously for 13 days and after 2 days of acclimation the experimental procedure began. The sensors (20 Hz) detected any movement of mice. Data were recorded by an IBM computer with dedicated software. No movements were detected by the sensors when mice were sleeping, inactive, or performed moderate self-grooming. Scores were obtained during 30-min intervals and expressed as counts per minute (cpm). The 24-h profile of activity was obtained by averaging 7 days of continuous registration. The position of Wt and Het mouse cages in the rack was equally distributed in rows and columns. Animals were provided with tap water and food pellets *ad libitum*. After the first 7 days of spontaneous activity, all animals were subjected to an injection of saline (a mild stressful stimulus), at 11 h (dark phase), and locomotor activity monitored up to 3 days later. The analysis of the locomotor profile over a period of 7 h (11–18) after saline injection was performed to evaluate the immediate stress response.

### Monoamines and their metabolites: HPLC determination

Subsequently to behavioral studies, male mice (*N* = 9 Wt, *N* = 10 Het) were sacrificed, their brains removed and rapidly dissected on ice to obtain the olfactory bulb, frontal cortex, striatum, Hip, and cerebellum for HPLC analysis. All samples were immediately flash frozen on dry ice, and then stored at −80°C until further processing. HPLC was performed according to Ref. (64). In particular, each brain region was weighed, ultrasonicated in 0.1 M perchloric acid, centrifuged for 20 min at 15,000 g (4°C) and the supernatant was used for monoamine neurotransmitters and their metabolites detection. The endogenous levels of 5-HT and 5-HT metabolite (5-hydroxyindolacetic acid; 5-HIAA), dopamine (DA) and final DA metabolite (homovanillic acid; HVA), and norepinephrine (NA) and NA metabolite (4-hydroxy-3-methoxyphenyl-glycol, MOPEG) were assayed by HPLC using a SphereClone 150 mm × 2 mm column (3-μm packing). Detection was accomplished with a Unijet cell (BAS) with a 6-mm-diameter glassy carbon electrode at +650 mV versus an Ag/AgCl reference electrode, connected to an electrochemical amperometric detector (INTRO, Antec Leyden, The Netherlands). For each analysis, a set of standards containing various concentrations of each compound (monoamines and their metabolites) was prepared in the perchloric acid solution, and calibration curves were calculated by a linear regression. The retention time of calibration standards was used to identify peaks, and areas under each peak were used to quantify monoamine levels. Results were normalized to the weight of wet tissue.

### Magnetic resonance imaging and spectroscopy

**Figure 1:**
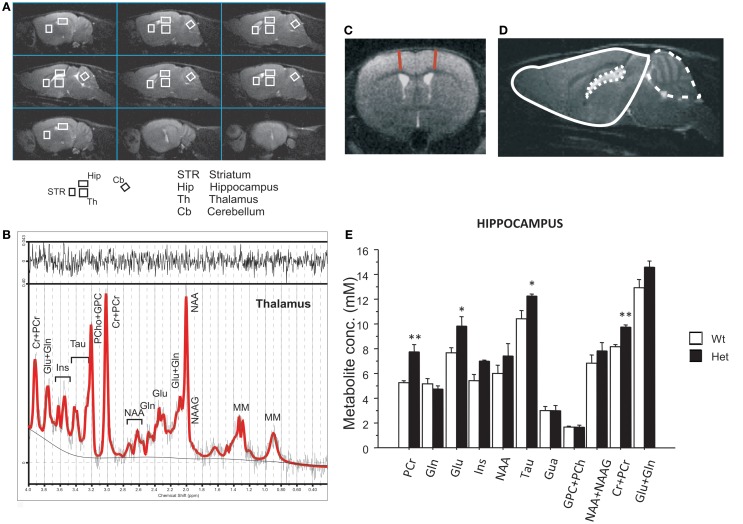
**Magnetic resonance imaging and spectroscopy performed in 4-month-old reeler mutant mice**. **(A)** MRI panel – example of *in vivo* sagittal T2-weighted spin-echo images (TR/TE = 3000/70 ms, slice thickness 0.8 mm, NS = 2, FOV = 20 mm × 20 mm, matrix 128 × 128). Voxels localized on STR, Hip, Th, and Cb are indicated by the white rectangles. **(B)** MRS panel – examples of *in vivo* 1H spectra (as a black trace), acquired from the thalamus (PRESS, TR/TE = 4000/23 ms, NS = 256). The result of LCModel fit is shown as a red trace superimposed on the spectrum. Metabolite assignments: Ins, inositol; Cr, creatine; PCr, phospho-creatine; Glu, glutamate; Gln, glutamine; Tau, taurine; PCho, phospho-choline; GPC, glicero-phospho-choline; NAA, *N*-acetyl-aspartate; NAAG, *N*-acetyl-aspartyl-glutamate; MM, macromolecules. **(C)** Examples of axial fast T2-weighted MR images from reeler heterozygous (Het) mice, respectively (TR/TEeff = 3200/60 ms, ns = 4, slice thickness 0.6 mm, 24 slices, matrix 256 × 256, FOV = 25 mm × 25 mm, which correspond to voxel resolution of 98 × 98 × 600 μm^3^). The red lines show the motor cortex thickness measure. **(D)** Example of brain segmentation for volumetric analyses of brain (plain line), cerebellum (dashed line), and ventricles (dotted line). **(E)** The histogram shows metabolite concentrations in hippocampus (Hip) for the two groups. Data are expressed as mean + SEM, *P < 0.05, **P < 0.005 between wildtype and heterozygous reeler mice. *N* = 7 Wt and *N* = 7 Het.

At 4 months of age, a separate cohort of male mice (*N* = 7 Wt, *N* = 7 Het), was subjected to *in vivo* MRI and magnetic resonance spectroscopy (MRS). During the MR analyses, animals were anesthetized with 2.5–2.0% isoflurane in oxygen 1 l/min (Isoflo, Abbott SpA, Latina, Italy). An integrated heating system allowed maintaining the animal body temperature at 37.0 ± 0.1°C. All MRI and MRS experiments were conducted on a 4.7 T Varian/Agilent Inova animal system (Agilent Inc., Palo Alto, CA, USA), equipped with actively shielded gradient system (max 200 mT/m, 12 cm bore size). A 6-cm diameter volume coil was used for transmission in combination with an electronically decoupled receive-only surface coil (Rapid Biomedical, Rimpar, Germany). Spin-echo sagittal anatomical images (TR/TE = 3000/60 ms, 13 consecutive slices of 0.8 mm thickness, FOV = 20 mm × 20 mm, matrix of 128 × 128, 2 averages) were acquired for accurate positioning the voxel for the MRS study. Single voxel localized ^1^H MR spectra (PRESS, TR/TE = 4000/23 ms, ns = 256 or 512) were collected from relevant brain areas: Hip (11.7 μl), striatum (STR, 10.4 μl), thalamus (Th, 12.96 μl), and cerebellum (Cb, 7.45 μl), as shown in Figure 1A and defined in the mouse brain atlas (65). Quantitative MRS protocol, including water T2 measurements, was applied (66) and T2 measurements were performed on water signal in order to identify any change in the mutant mice. Unsuppressed water signal was used for metabolite quantification (assuming 79.9% for gray matter water content). Spectra were analyzed using LCModel (67). Only those metabolites that were estimated to have Cramer–Rao lower bounds (CRLB) <20%, which corresponded to an estimated concentration error <0.2 μmol/g, were included into the quantitative analysis. In some cases, metabolites that have resonance overlapped or very close are also given as their sum. An example of spectra and its LCModel analysis is shown in Figure 1B.

Multislice fast spin-echo axial images (TR/TEeff = 3200/60 ms, ns = 4, slice thickness 0.6 mm, 24 slices, matrix 256 × 256, FOV = 25 mm × 25 mm, which correspond to voxel resolution of 98 × 98 × 600 μm^3^) were also acquired for volumetric analyses.

Motor cortex thickness was measured at +1.32 from bregma as shown in Figure 1C. Volumetric analyses of the whole brain have been performed from olfactory bulb to cerebellum excluded. Ventricles and cerebellum volumes were also measured. Brains were manually segmented for forebrain, ventricles, and cerebellum using Varian/Agilent Imaging Browser, which perform a 3D-volume calculation by summing the pixels areas on the center of each slices and interpolating the cross sectional areas between the center of the other slices (Agilent Inc., Palo Alto, USA) on MR images. Manual segmentation of the ventricles was facilitated by the high contrast that cerebrospinal fluid has in the MR images. Figure 1D shows an example of segmentation (slice central to the brain in sagittal images).

### Statistical analysis

A mixed-model ANOVA with repeated measures was used to analyze: (1) sniffing of different body areas (anogenital, body, or head), (2) spontaneous locomotor activity in the home-cage, (3) number of USVs for each minute of interaction, and (4) probability of vocalizations in eight call categories with genotype as between-subject factor. Probability of vocalizations within each genotype was calculated as number of calls in each category for each subject/total number of calls analyzed in each subject and standardized by angular transformation.

Data relative to MRI and MRS were analyzed by a one way ANOVA with genotype as the independent factor and MRI/MRS parameters (values of water T2, metabolite levels in each brain region and volume of each brain region) as dependent factor. Differences between genotypes in each brain region with respect to serotoninergic, dopaminergic, and noradrenergic systems (5-HT, 5-HIAA, and 5-HT turnover for serotoninergic system; DA, HVA, DOPAC, and DA turnover for dopaminergic system; and NA, MOPEG, and NA turnover for noradrenergic system) were determined by a multivariate analysis of variance (MANOVA), due to the potentially high correlation between these dependent variables within each system. Pillai’s statistic was used. Univariate ANOVAs were conducted for each variable (Statview II, Abacus Concepts, CA, USA).

For all comparisons, data are expressed as mean ± SEM and significance was set at *P* < 0.05. *Post hoc* comparisons were performed using Tukey’s test only when a significant *F*-value was determined.

## Results

### Male–female social interaction test

**Figure 2:**
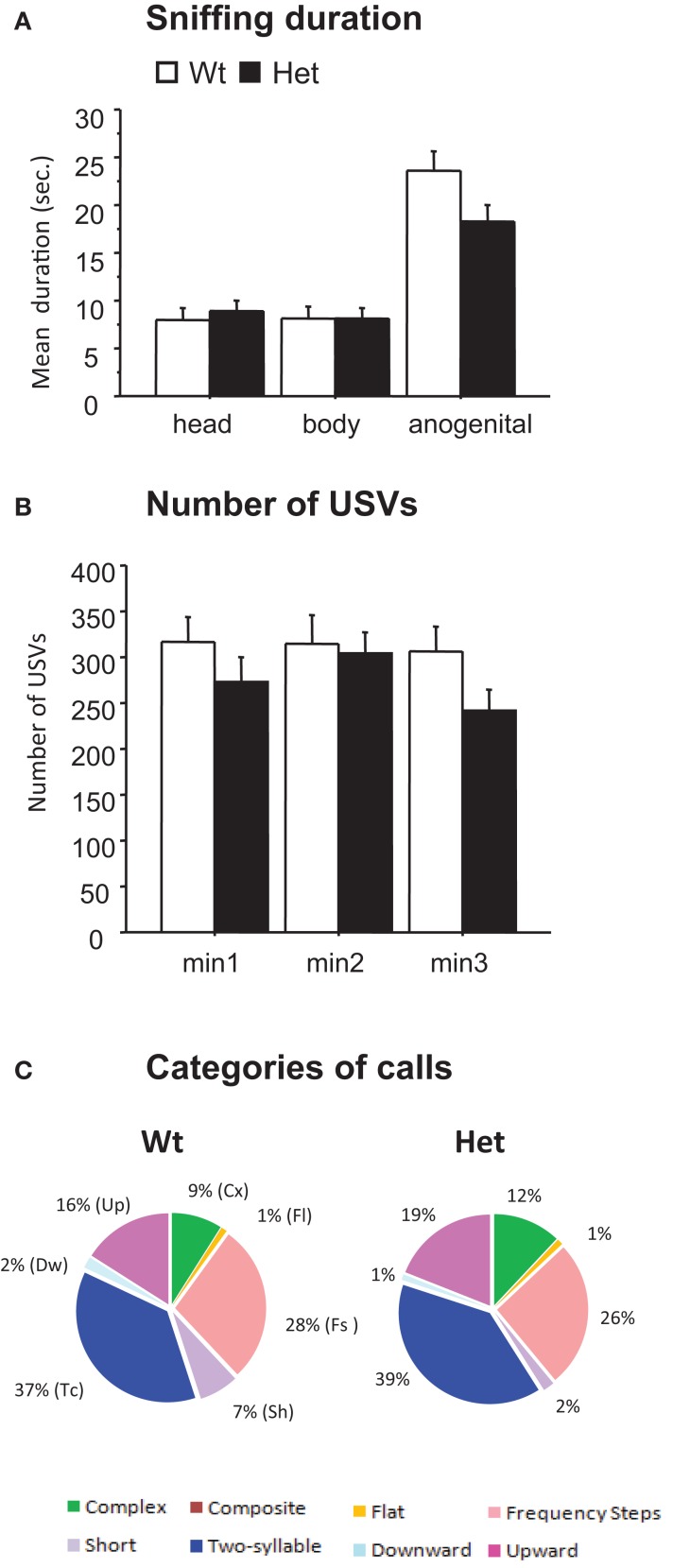
**Male–female social interaction test (3-min-session) performed in 3-month-old reeler mutant mice**. Parameters measured during a direct interaction between a male with a sexually receptive C57BL/6J female. **(A)** Sniffing duration. **(B)** Number of ultrasonic vocalizations. Data are expressed as mean + SEM. **(C)** Pie graphs show the percentages of the different call categories emitted by Wt and Het reeler mice. Percentages were calculated in each genotype as number of calls in each category for each subject/total number of calls analyzed for each subject. Number of calls analyzed: 17195 in Wt and 8454 in Het. *N* = 9 Wt and *N* = 21 Het.

To assess the presence or absence of a social communication deficit in Het reeler mice, we evaluated the behaviors and the USVs emitted by a male mouse during the interaction with an estrus C57BL/6J female. Analysis of the social sniffing response on different body areas (head, body, and anogenital) did not reveal significant effects of genotype [frequency, *F*(2,56) = 0.15, *P* = 0.858, (data not shown); duration, *F*(2,56) = 1.74; *P* = 0.183, Figure 2A]. No genotype effect was detected on explorative behaviors such as *rearing* [frequency, *F*(1,28) = 0.27, *P* = 0.610 and duration, *F*(1,28) = 0.30, *P* = 0.589] and *digging* [frequency, *F*(1,28) = 0.75, *P* = 0.392 and duration, *F*(1,28) = 1,15, *P* = 0.292] (data not shown).

Analysis of the USVs emitted by male mice during the social interaction test did not detect significant differences between Het reeler and Wt mice: number of USVs [number of calls × genotype, *F*(2,56) = 0.89, *P* = 0.41, Figure 2B], mean duration [*F*(2,54) = 0.79, *P* = 0.457 (data not shown)], peak frequency [*F*(2,54) = 0.43, *P* = 0.650 (data not shown)], and peak amplitude [*F*(2,54) = 0.14, *P* = 0.863 (data not shown)]. As a whole, the pattern of sonographic structures did not differ between Het reeler and Wt mice indicating a comparable vocal repertoire in both genotypes (see pie graphs in Figure 2C).

### Locomotor activity in the home-cage

Sleep problems and irregular sleep–wake cycles have been identified in several ASD children (68–71). Alterations in circadian rhythm lead to anxiety-like, impulsive, and depressive behaviors both in humans and mice (72–74). In the present study, we evaluated baseline circadian locomotor activity in the home-cage as well as response to a mild stressful stimulus represented by a saline injection to check for abnormalities in the spontaneous locomotor activity that could affect the behavioral performances.

**Figure 3:**
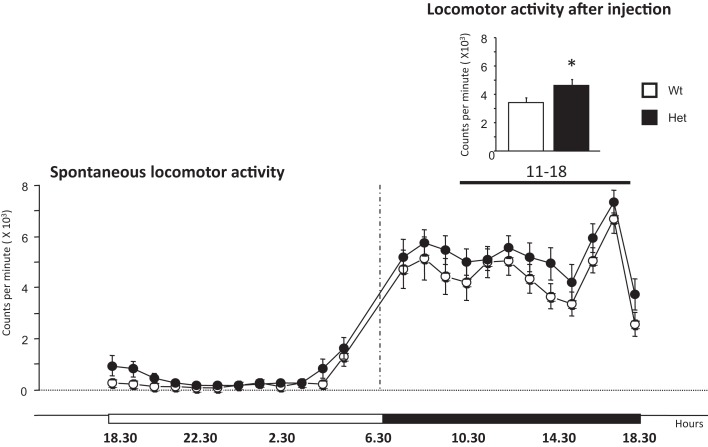
**Locomotor activity expressed spontaneously in the home-cage by 6-month-old wildtype and heterozygous reeler male mice**. The main graph indicates spontaneous locomotor activity monitored for 1 week. The graph on the top right shows the spontaneous locomotor activity monitored for 7 h after a mild stressful stimuli represented by a saline injection at 11 h (dark phase). Data are expressed as mean ± SEM, *P < 0.05, between wildtype and heterozygous reeler mice. *N* = 9 Wt and *N* = 10 Het.

Analysis of spontaneous locomotor activity measured in the home-cage for 7 days revealed, as expected, an increased activity in mice of both genotypes during the dark phase of the light/dark cycle [phase effect, *F*(1,17) = 239.05, *P* < 0.001] (see Figure 3). No genotype differences were found [light phase: genotype, *F*(1,17) = 2.99, *P* = 0.102; dark phase: genotype, *F*(1,17) = 1.34, *P* = 0.263].

The analysis of the locomotor profile over a period of 7 h after saline injection (11–18, dark phase) was performed to evaluate the immediate stress response. Het reeler mice increased significantly their locomotor activity as compared to Wt mice [genotype, *F*(1,17) = 5.63, *P* = 0.029] thus revealing a genotype-dependent increased sensitivity to mild stress challenge (see graph on the top right of Figure 3). After this 7-h-period, locomotor activity goes back to the normal activity profile [genotype effect, *F*(1,17) = 0.226, *P* = 0,64; genotype × hours, *F*(1,23) = 0.38, *P* = 0.99; data not shown].

### Monoamines and their metabolites: HPLC determination

High performance liquid chromatography determination has been applied to investigate whether *reelin* mutation influences the different monoamine systems. MANOVA revealed a significant genotype effect on several components of the dopaminergic system in hypothalamus [Pillai’s Trace: *F*(4,13) = 9.73, *P* < 0.001] and in Hip [Pillai’s Trace: *F*(4,13) = 4.97, *P* = 0.005]. ANOVA showed a strong reduction of DA levels [genotype effect, *F*(1,16) = 5.29, *P* = 0.035] and a consequent increase in the DA turnover [genotype effect, *F*(1,16) = 7.43, *P* = 0.015] in the cortex of Het reeler as compared to Wt mice (see Table 1). Moreover, Het reeler mice showed an increase of DOPAC and HVA levels [genotype effect: DOPAC, *F*(1,16) = 18.88, *P* < 0.001; HVA, *F*(1,16) = 11.47, *P* = 0.003] in hypothalamus. ANOVA evidenced also a decrease of HVA levels [*F*(1,16) = 6.43, *P* = 0.022] and an increase of DA turnover [genotype effect, *F*(1,16) = 6.14, *P* = 0.025] in Hip of Het mice.

**Table 1:** **Levels of monoamines and their metabolites detected *ex vivo* in cortex, bulbs, hypothalamus, striatum, hippocampus, and cerebellum (mean ± SEM: picogram per milligram of wet tissue)**.

Brain region	Genotype		Neurotransmitter, metabolite concentration picogram per milligram wet brain weight)				
		Noradrenergic system		Dopaminergic system			Serotoninergic system	
		NA	MOPEG	DA	DOPAC	HVA	5-HT	5-HIAA
Cortex	Wt	469.60 ± 22.99	102.74 ± 7.53	874.14 ± 268.95	134.50 ± 19.85	252.97 ± 35.58	523.10 ± 29.72	341.42 ± 17.59
	Het	440.84 ± 28.76	102.47 ± 6.20	296.37 ± 69.32*	108.65 ± 18.65	184.52 ± 40.76	470.09 ± 31.15	285.37 ± 14.32
Bulbs	Wt	291.82 ± 24.28	46.35 ± 5.94	312.11 ± 33.66	100.26 ± 8.63	152.87 ± 15.49	247.65 ± 25.63	191.88 ± 16.88
	Het	346.10 ± 14.98	66.26 ± 6.28	396.15 ± 23.84*	102.06 ± 6.83	154.78 ± 8.95	131.53 ± 20.97	192.13 ± 9.76
Hypothalamus	Wt	1789.74 ± 80.36	205.08 ± 23.06	394.87 ± 16.83	116.63 ± 6.12	362.10 ± 13.19	518.33 ± 22.43	934.82 ± 42.78
	Het	1742.52 ± 81.41	183.74 ± 7.50	540.86 ± 163.65	144.44 ± 5.48**	444.55 ± 13.27**	487.36 ± 29.79	864.22 ± 19.73
Striatum	Wt	129.71 ± 32.78	339.05 ± 49.89	12521.13 ± 1661.54	2718.95 ± 326.72	4310.08 ± 383.59	453.07 ± 30.88	654.30 ± 23.09
	Het	128.12 ± 19.16	261.70 ± 25.90	12275.97 ± 1376.40	2847.63 ± 196.75	4583.34 ± 398.19	109.34 ± 26.48	679.84 ± 24.65
Hippocampus	Wt	508.02 ± 33.64	115.37 ± 6.77	82.16 ± 7.61	18.21 ± 1.33	69.47 ± 8.48	505.52 ± 61.78	540.99 ± 37.90
	Het	423.67 ± 39.12	108.17 ± 8.75	84.80 ± 13.48	25.16 ± 4.81	42.01 ± 6.93*	580.54 ± 33.18	503.31 ± 53.05
Cerebellum	Wt	402.46 ± 28.48	48.85 ± 3.12	5.09 ± 1.06	12.52 ± 2.38	80.66 ± 5.09	115.60 ± 17.51	129.05 ± 6.03
	Het	433.56 ± 17.08	46.17 ± 3.42	6.75 ± 2.68	18.77 ± 7.55	79.52 ± 3.59	118.94 ± 21.13	126.85 ± 6.04

**Brain region**	**Genotype**	**Neurotransmitter turnover**						
		**NA**	**DA**	**5-HT**				

Cortex	Wt	0.22 ± 0.01	0.24 ± 0.05	0.66 ± 0.03				
	Het	0.24 ± 0.01	0.45 ± 0.05*	0.62 ± 0.03				
Bulbs	Wt	0.16 ± 0.02	0.33 ± 0.02	0.80 ± 0.05				
	Het	0.19 ± 0.01	0.26 ± 0.02*	0.86 ± 0.05				
Hypothalamus	Wt	0.11 ± 0.01	0.30 ± 0.02	1.81 ± 0.09				
	Het	0.11 ± 0.01	0.35 ± 0.04	1.83 ± 0.11				
Striatum	Wt	4.09 ± 1.01	0.23 ± 0.03	1.47 ± 0.06				
	Het	2.86 ± 0.7E	0.26 ± 0.03	1.71 ± 0.10				
Hippocampus	Wt	0.23 ± 0.01	0.23 ± 0.02	0.95 ± 0.11				
	Het	0.26 ± 0.02	0.29 ± 0.02*	0.87 ± 0.07				
Cerebellum	Wt	0.12 ± 0.01	2.83 ± 0.51	1.25 ± 0.13				
	Het	0.11 ± 0.01	6.76 ± 2.45	1.36 ± 0.23				

*P < 0.05, **P < 0.005 between wildtype and heterozygous reeler mice.

Heterozygous mice showed an higher DA levels [genotype effect, *F*(1,16) = 4.38, *P* = 0.052] and a lower DA turnover in olfactory bulb [genotype effect, *F*(1,16) = 6.87, *P* = 0.018] than Wt mice. No genotype related differences were found on noradrenergic and serotoninergic systems in each brain areas analyzed.

### Magnetic resonance imaging

To acquire deeper information into the functional state of brain areas involved in ASD, we assessed a ^1^H magnetic resonance in adult reeler male mice. Enlarged ventricles and reduced cerebellum are typical features of reeler mice (75). Volumetric analyses confirmed a cerebellum reduction [genotype effect, *F*(1,10) = 15.50, *P* = 0.002] and an enlargement of ventricles volume [genotype effect, *F*(1,11) = 8.01, *P* = 0.016] in Het reeler when compared to Wt mice. No genotype differences were detected in volume [genotype effect, *F*(1,11) = 0.86, *P* = 0.374] and medial motor cortex (MC) thickness [genotype effect, *F*(1,11) = 3.62, *P* = 0.083] (see Table 2).

**Table 2:** **Analysis for forebrain, cerebellum, and ventricles volume**.

	Forebrain volume (μ1)	Ventricles volume (μ1)	Cerebellum volume (μ1)	Medial cortex thickness (mm)
Wt	366.17 ± 2.6	3.29 ± 0.5	59.18 ± 1.2	1.16 ± 0.1
Het	369.85 ± 3.0	5.25 ± 0.4*	53.03 ± 1.1*	1.22 ± 0.1

Data are expressed as mean ± SEM. *P < 0.05, between wildtype and heterozygous reeler mice.

### Magnetic resonance spectroscopy

To investigate the possible alterations in brain metabolism of adult reeler male mice, we performed MRS. The high quality spectra allowed reliable quantification (%SD <20%) not only for the commonly observed *N*-acetyl-aspartate (NAA), total creatine (Cr + PCr) and total choline resonances (NAA + NAAG), but also for glutamine (Gln), glutamate (Glu), taurine (Tau), and inositol (Ins) in all the investigated brain regions.

Water T2 analyses confirmed that no changes between the genotypes occurred in the T2s in Hip [*F*(1,10) = 2.52, *P* = 0.143], Striatum [*F*(1,10) = 0.02, *P* = 0.883], Thalamus [*F*(1,10) = 1.15E-5, *P* = 0.977], and Cerebellum [*F*(1,10) = 0.32, *P* = 0.584] (data not shown).

Metabolic changes were detected in Hip while no differences have been found for any metabolite in thalamus, striatum, and cerebellum. Het reeler mice showed increased levels of Glu [genotype effect, *F*(1,11) = 4.61, *P* = 0.044], Tau [genotype effect, *F*(1,11) = 4.82, *P* = 0.050], PCr [genotype effect, *F*(1,7) = 18.08, *P* = 0.003], and total amount of PCr + Cr [genotype effect, *F*(1,11) = 24.68, *P* < 0.001] in Hip as compared to Wt mice (see Figure 1E).

## Discussion

Reelin is a glycoprotein playing a crucial role during development: it regulates neuronal migration and brain lamination (6, 8, 29, 30, 76, 77) and its reduced or complete lack of signaling impairs neuronal connectivity and synaptic plasticity (43, 78). Moreover, recent data suggest that a defect in reelin signaling pathway confers greater susceptibility to autism (20–25, 27).

For these reasons, we consider Het reeler mice, haploinsufficient for *reelin* and sharing some neurochemical and behavioral features with autistic patients, a suitable animal model for studying the effects of *reelin* deficiency in determining social communication deficits and in changing brain monoamine and brain metabolites levels. Unfortunately, no comparison can be drawn with homozygous mutant mice, since adult knockout reeler mice did not survive longer than weaning (79–82).

### No deficits in social and vocal repertoires during courtship

To our knowledge, this is the first time that a detailed analysis of the adult male vocal repertoire has been performed in this mutant line. Only behavioral data on same-sex interactions or approaching/recognizing a conspecific have been collected (44, 51, 52).

Recently, we characterized vocal and motor repertoires on homozygous and Het reeler pups (60) evidencing a general delay in vocal and motor development during the first 2 weeks of postnatal life, in line with the alterations in the same two systems observed in children with ASD. In addition, a preferential use of a specific call category (two-components) at pnd 2 and 6 was detected in both mutants (Het and homozygous), whereas an increased number of vocalizations characterized only Het pup’s emission.

Contrary to what we found in pups, adult Het male mice did not show deficits on USVs emitted during courtship of a female in estrous. Social behaviors, generally associated to this vocal emission, were not affected either. These results are in contrast with the reduction in anogenital sniffing and/or the number of USVs found in other ASD animal models such as BTBR, En2, NMDA-Nr1, NLG3, NLg4, Dlg4, and FmR1 mice (53, 83–87), but in line with data collected on Shank3 mice, carrying a mutation strongly implicated in autism and Phelan-McDermid 22q13 deletion syndrome, where male knockout mice did not present alterations in social communication and interaction (88).

These data thus confirm that adult Het reeler mice present deficits on cognitive performances but not on social domains (44–47, 49–52). It is worth of notice that intellectual disabilities are present in about 50% of autistic individuals. Due to the cerebellar alteration leading to death shortly after weaning, no data could be collected on mice with the complete deletion in the reelin gene, thus we cannot exclude an impairment of the social domain only related to the complete deletion of *reelin*.

### Over response to a mild stress stimuli

Previous studies indicated that Het reeler mice have several abnormalities in their brain architecture (40–42), but, at a first sight, their phenotype is absolutely “normal” (7, 44, 89). Some behavioral deficits become evident only after a “second hit” (7, 63, 90, 91) supporting the “double-hit” theory postulating a gene–environment interaction in the pathogenesis of several neurodevelopmental disorders such ASD (89). Depending on the features of environmental factors and the time-window of insult interacting with reelin expression, an individual could thus develop one neurodevelopmental disorder rather than another one (i.e., schizophrenia versus ASD).

Our previous evidence shows that either an environmental pollutant or, for example, an activated stress reactivity caused by repeated separation from the dams, elicits different responses as a function of the mouse genotype (91). In line with these data, in the present study, no significant genotype differences were found in basal activity levels of mice monitored in their home-cages for 1 week. By contrast, after a saline injection (a mild stressful stimulus), the Het male displayed a higher locomotor activity profile as compared to Wt male mice. Already in a previous study, our group showed a hyperactive profile in Het adolescent reeler mice following handling plus saline injections (63). Altogether, these data indicate that Het reeler mice show a different response to environmental stimuli, confirming the suitability of such mutant line for the study of gene–environment interactions (7, 92).

Moreover, a deficit in behavioral inhibition has been reported as a core alteration of Het reeler mice, associated with dysfunctions of mesolimbic DA transmission (93) and reduced GABAergic transmission in central nervous system (40, 94–96).

### Impairment in the dopaminergic pathway

To correlate observed behavioral abnormalities to the neural systems reportedly affected by *reelin* mutation, we conducted HPLC analyses in different brain areas involved in autism, detecting impairments in the dopaminergic system. Specifically, Het reeler mice had decreased DA levels in cortex and increased levels in the olfactory bulb, whereas DA turnover was altered in cortex, bulb, and Hip.

A disruption of DA maturation in *reelin* haploinsufficient mice had been already suggested: a reduced locomotor activation by d-amphetamine in reeler mice was associated with an exaggerated drug-induced stereotyped behavioral syndrome (90). Moreover, Ballmaier et al. (93) reported alterations in the mesolimbic DA pathway of Het reeler mice. In particular, they found that Het mice exhibit a reduction in DA transporter immunoreactivity and D2 receptor mRNA in the limbic striatum and the ventral tegmental area (93). In agreement with our study, they did not find any significant alteration in the dopaminergic markers examined in the nigrostriatal pathway of Het reeler mice.

Alterations in DA levels and its turnover have been found in brain areas primarily associated to reward. Individuals with ASD show reduced responsiveness to reward stimuli, a feature that appears to be especially prominent with social reinforces such as facial expressions, spoken language, and gestures (97, 98). No effects have been found in the striatum where DA contributes to motor performances.

In addition, the neurotransmitter DA plays a pivotal modulatory role on executive functions and learning (99, 100), thus a dysfunctional DA system could underlying the cognitive deficits detected in Het mice.

### Glutamate and taurine increased levels in hippocampus

To gain deeper insights into the functional state of brain areas involved in ASD, we carried out a ^1^H MRI guided spectroscopy examination in adult reeler mice. MRS is a powerful, non-invasive tool for monitoring neurological diseases (101) and it is also used in clinical studies on autistic individuals (102). Abnormalities in neurotransmitter pathways have been associated to ASD, with evidence for a possible implication of glutamatergic, GABAergic, and serotonergic imbalances (102).

In the Hip, as compared to Wt in Het mice, MRS showed increased levels of glutamate, taurine, phospho-creatine, and of the total amount of phospho-creatine plus creatine. Glutamate is the main brain excitatory neurotransmitter involved in cognitive functions, although in excessive quantities can cause neuronal damages typical of neurodegenerative diseases (for example, Alzheimer’s and Huntington’s diseases) (103–105). The higher levels of glutamate in the Hip of Het reeler mice are in agreement with previous findings reporting an increase of glutamate in Hip (106) and cortex (107) of ASD patients; altogether these evidences support the hypothesis of an imbalance between excitatory and inhibitory (GABA) systems as one of the possible causes of autism (107).

Recently, clinical trials with glutamate antagonists have been initiated, since they have been proved to be effective in rescuing social deficits and repetitive behaviors in selected animal models of autism (108). Also the presence of high levels of taurine in the Hip could be correlated to high levels of glutamate. In fact, taurine appears to have a protective action against glutamate excitotoxicity (109) and it is widely considered a general index of neuronal functionality.

The largest meta analysis performed on ASD patients showed evidence that ASD is characterized by age-dependent fluctuations in metabolite levels across the whole brain. In particular, significant reduction in the level of a cerebral metabolites, NAA, a specific neuronal marker, in whole gray matter of ASD children as well as significant increase in the total pool of creatine (phospho-creatine plus creatine) in adult subjects were observed (110). The observed differences in creatine as a function of age and brain region, suggest caution in the use of Cr-based ratio measures of metabolites. For this reason, we adopt a quantitative approach for brain metabolites level determination, which has been validated on phantom (111) as well as on other animal models of psychiatric and neurodevelopmental diseases (51, 112–114).

## Conclusion

In the literature, Het reeler mice are widely considered a reliable animal model of either autism or schizophrenia. Genetic and molecular evidences showed that reelin messenger-RNA and its protein are downregulated in cortical, hippocampal, and cerebellar neurons of patients suffering of schizophrenia and autism (3, 8, 10, 20, 115, 116). In particular, these mutant mice are characterized by decreased contextual fear conditioning (48), prepulse inhibition (43, 117), impaired executive functions (45), and associative learning (48), all typical traits of schizophrenia. In addition, Het reeler mice yielded autistic-like deficits in social behavior and communication in the first two postnatal weeks of age (60) and perseverative (51) and hyperactive behaviors (44) at adulthood. Discordant evidences exist on this model, possibly associated with differences in the genetic background, age of mice, training and testing protocols, and rearing conditions (52, 89).

Overall, our results, together with data previously collected by our (Laviola and collaborators) and other groups suggest that Het reeler mice have common behavioral traits to both these neurodevelopmental disorders. Moreover, these studies indicate the suitability of this mutant line to investigate the role of *reelin* as vulnerability factor on the etiology of both disorders. In addition, Het reeler mutant mice may represent a useful animal model to develop novel treatment strategies for these devastating human disorders. For example, our HPLC and MRS results favor further studies to evaluate the effects of DA agonist or glutamate antagonist treatments on behavioral and neurochemical responses.

## Conflict of Interest Statement

The authors declare that the research was conducted in the absence of any commercial or financial relationships that could be construed as a potential conflict of interest.
